# Site-specific neoepitope induction by RNA editing reprograms tumor immunogenicity

**DOI:** 10.3389/fimmu.2026.1839930

**Published:** 2026-06-09

**Authors:** Shangyuan Liu, Chunxiu Dong, Lin Chen, Jiahong Yan, Xiaoshan Luo, HaiChao Huang, Zonghao Qiu, Xuhong Gan, Jiali Yang, Jiafeng Zhu, Chengjie Xiong, Lihong Huang, Linlin Wang, Zhengning Yang, Zhongxuan Meng, Senrong Yi, Yuxin Chen, Chijian Zuo, Qibin Leng, Xiaoling Wang

**Affiliations:** 1 Guangzhou Medical University-Guangzhou Institutes of Biomedicine and Health (GMU-GIBH) Joint School of Life Sciences, Guangdong Provincial Key Laboratory of Protein Modification and Disease, The Guangdong-Hong Kong-Macao Joint Laboratory for Cell Fate Regulation and Diseases, Guangzhou Medical University, Guangzhou, Guangdong, China; 2 Suzhou CureMed Biopharma Technology Co., Ltd., Suzhou, China; 3 State Key Laboratory of Respiratory Disease, Guangzhou Institute of Cancer Research, The Affiliated Cancer Hospital, Guangzhou Medical University, Guangzhou, Guangdong, China

**Keywords:** RNA editing, neoantigen, ADAR, epitope, tumor immunogenicity

## Abstract

**Introduction:**

Neoantigens derived from somatic mutations exhibit high immunogenicity and specificity. However, their stochastic nature necessitates personalized vaccine design, and cancers with low tumor mutational burden often have insufficient neoantigen availability, limiting their clinical applicability. Here, we introduce RNA Editing-Induced Site-specific Neoantigen (REISN) as a strategy to remodel tumor immunogenicity by targeting predefined epitopes in widely expressed cancer/testis and embryonic antigens, including murine P1A and human AFP.

**Methods:**

We leveraged an MS2 coat protein (MCP)-ADAR-based A-to-I RNA editing system to selectively modify T cell receptor (TCR)-contact residues in major histocompatibility complex class I (MHC-I)-presented peptides derived from P1A and AFP. We further evaluated the immunogenicity and antitumor efficacy of lipid nanoparticle-formulated P1A-REISN circular RNA vaccination in immunocompetent BALB/c mice bearing tumors with P1A-REISN induction.

**Results:**

Site-directed RNA editing generated REISN-modified epitopes capable of activating REISN-specific naïve T cells. Vaccination with lipid nanoparticle-formulated P1A-REISN circular RNA elicited potent antigen-specific CD8+ T cell responses and suppressed tumor growth in immunocompetent BALB/c mice.

**Discussion:**

These findings provide proof-of-concept that site-directed RNA editing can be used to reprogram tumor immunogenicity by generating defined neoantigen-like epitopes from shared tumor-associated antigens. This strategy may expand neoantigen-based immunotherapy beyond tumors with high mutational burden and warrants further investigation for clinical translation.

## Introduction

Cancer immunotherapy has been recognized as one of the most transformative breakthroughs in oncology (1). Tumor-associated antigens (TAAs), such as cancer-testis antigens (CTAs; e.g., MAGE-A3 in up to 76% of melanomas and 55% of non–small cell lung cancers) and oncofetal antigens (e.g., AFP in 60-80% of hepatocellular carcinomas), remain key immunotherapy targets due to their broadly reactivation across malignancies yet restricted to immune-privileged tissues (e.g., testis and placenta) or embryogenesis (2–5). While their pan-cancer prevalence and tumor-selective profiles make them attractive targets for adoptive T cell therapies (e.g., TCR-T) and cancer vaccines, both classes face central tolerance (3, 5, 6).

Neoantigens, arising from genetic instability that gives rise to non-synonymous somatic mutations, have recently emerged as a pivotal focus in immuno-oncology due to their unique tumor-restricted expression profiles and potent immunogenicity (2). Accumulating evidence indicates that neoantigen, especially clonal neoantigen-directed T cell responses underpin the clinical efficacy of immune checkpoint inhibitors, providing a mechanistic link between mutational burden and therapeutic outcomes (7–10). Capitalizing on this paradigm, neoantigen vaccines have been developed to directly engage these mutation-derived epitopes and have demonstrated remarkable clinical promise (11–17). Nevertheless, the clinical implementation of personalized neoantigen vaccines faces three major limitations (1): insufficient neoantigen burden in subsets of patients with low tumor mutational load (2), suboptimal accuracy of current neoantigen prediction algorithms, and (3) prohibitive costs associated with individualized manufacturing (18–20). Compounding these challenges, Luksza et al. revealed an evolutionary immune evasion mechanism wherein tumors progressively downregulate or genomically eliminate immunodominant neoantigens during disease progression (21).

We present a rationally designed RNA-Editing Induced Site-specific Neoantigen (REISN) strategy that exploits programmable RNA base editing to engineer immunogenic neoepitopes at predefined TAA loci (Scheme 1). This strategy integrates three synergistic components (1): Reversible RNA-level editing through adenosine deaminases acting on RNA (ADARs)-mediated site-directed A-to-I conversion (22) (2); Pan-cancer targeting and tumor-specificity by targeting widely shared TAAs (3); Epitope reprogramming through selective modification of T-cell receptor (TCR)-contact residues on TAAs, converting self-tolerant epitopes into immunogenic neoepitopes while preserving MHC anchoring motifs.

Mechanistically, RNA editing, a naturally occurring post-transcriptional modification process, enables precise nucleotide conversion without altering the genomic DNA (22, 23). Adenosine-to-inosine (A-to-I) editing, mediated by adenosine deaminases acting on RNA (ADARs), constitutes nearly 90% of all RNA editing events in humans and dynamically regulates transcriptomic diversity across millions of genomic loci (22, 23). During ribosomal translation, inosine is decoded as guanosine, effectively inducing site-specific A-to-G substitutions (24). This recoding capacity allows for the generation of non-synonymous amino acid changes at strategic loci within TAAs, thereby converting self-antigens into immunogenic neoepitopes. The endogenous expression of ADAR in human cells provides a biocompatible catalytic scaffold, enhancing the translational feasibility of REISN (25–27). While RNA editing technologies, including CRISPR-Cas13 systems, endogenous ADAR-recruiting platforms (LEAPER and RESTORE), and chemically engineered guide RNA-oligonucleotides, have demonstrated therapeutic potential for correcting pathogenic mutations in monogenic disorders, their application in oncology remains largely unexplored (28–36). Here we provide proof-of-concept that RNA editing technology can be used to create REISN epitopes targeting predefined TCR-contact residues on MHC class I-presented peptides of TAA.

## Methods

### Cell lines and plasmids

Cell lines were cultured in Roswell Park Memorial Institute (RPMI)-1640 (A549, Jurkat CD8-NFAT and HepG2) or DMEM (293T, A375, CT26 and HeLa), supplemented with 10% fetal bovine serum (Lonsera) and 1% penicillin/streptomycin (Life Technologies). HeLa cells were purchased from Meisen Chinese Tissue Culture Collections (Zhejiang, China), while the others were obtained from the American Type Culture Collection (ATCC). Monthly PCR-based mycoplasma tests were conducted to ensure that all cell lines used were mycoplasma-free.

The Jurkat CD8-NFAT (JK8NF) reporter cell line was used to generate JK8NF-TCR041 cells as previously described (37). JK8NF cells were transduced at a MOI of 10 with the lentivirus packaged with pLKO-TCR041 and confirmed by human CD3 surface expression. CT26-arP1A and CT26-arNT cell lines were generated by transducing wild-type CT26 cells with the lentivirus encoding P1A (pLKO.1-P1A selected by 500 μg/mL hygromycine B for 7 days) and mADAR (pCW57-ADAR selected by 10 μg/mL puromycin for 7 days) sequence at a MOI of 2, followed by transduction and selection of pLKO-arP1A or pLKO-arAFP lentivirus at a MOI of 10 using 10 μg/mL Blasticidin S HCl for 7 days. pCW57-MCS1-P2A-MCS2 (Neo) was a gift from Adam Karpf (Addgene plasmid # 89180; http://n2t.net/addgene:89180; RRID: Addgene_89180). pLKO.1 puro was a gift from Bob Weinberg (Addgene plasmid # 8453; http://n2t.net/addgene: 8453; RRID: Addgene_8453). Primers used for arRNA and target gene expression were listed in **Supplementary Table 1**, Supporting information.

CT26-P1A-WT and CT26-P1A-Y37C were generated by transducing wild-type CT26 cells with the lentivirus encoding P1A-WT or P1A-Y37C at a MOI of 10. A375-AFP-WT and A375-AFP-K161R cell lines were generated by transducing wild-type A375 cells with the lentivirus encoding AFP-WT or A375-AFP-K161R at a MOI of 10, followed by sorting with the FACSAria III (purity >95%).

### Flow cytometry

Fluorochrome-labeled antibodies were listed in **Supplementary Table 2**, Supporting information. Peptide-MHC monomers were produced in-house following protocols from the NIH Tetramer Core Facility. APC and PE-labeled tetramers were generated by conjugating biotinylated peptide-MHC monomers with streptavidin–phycoerythrin (PE) (BD Biosciences, cat 554061) or streptavidin-allophycocyanin (APC) (Miltenyi Biotec, cat 130-106-791) at a 4:1 molar ratio. All antibodies and tetramers were used at 1:200 dilution. Data were quantified using BD FACSAria III flow cytometer (BD Biosciences) and CytoFLEX S (Beckman Coulter) and analyzed with FlowJo V10 and CytExpert software.

### Transfection and RNA editing efficiency assay

Hela cells and 293T cells were transiently transfected with 250 ng of pUC57-arRNA, 25 ng of pcDNA3.1-MCP-ADAR1 and 12.5 ng of target gene expression plasmid using Lipo8000 reagent at 50% confluence (Beyotime, C0533) following the manufacturer’s instructions. Cells were harvested 48 hours later for subsequent RNA extraction and editing efficiency analysis by reverse transcription PCR and Sanger sequencing. Total RNA of tumor cells was extracted using the RaPure Total RNA Micro Kit (Magen, R4012-03) and reverse-transcribed using a HiScript III RT SuperMix for qPCR (Vazyme, R323-01). Negative control reactions without reverse transcriptase were performed in all RT-PCR experiments to exclude genomic DNA contamination. The cDNA was used as the template for the subsequent RT-qPCR analysis using ChamQ Universal SYBR qPCR Master Mix (Vazyme, Q711-02).

### RNA editing efficiency analysis

RNA was isolated from cultured tumor cells and bulk tumor tissue using the RaPure Total RNA Micro Kit (Magen, R4012-03). RNA purity was assessed using NanoDrop and its integrity was evaluated using Agilent 4200 TapeStation. Sequence files were analyzed by Chromas software. A to I(G) editing efficiency was analyzed by using QSVanalyser software and calculated as the peak height of G/(A + G)×100%.

### Tumor cell coculture

TCR041-NFAT-Jurkat-CD8 cells and A375 or 293T cells were seeded at the indicated E:T ratio in 96-well plates (Thermo) at a density of 2×10^4^ cells per well. Plates were incubated at 37 °C with 5% CO_2_ for 48 hours. The TCR041-NFAT-Jurkat-CD8 cells were harvested and analyzed by flow cytometry.

### Isolation of tumor-infiltrating lymphocytes

TILs were isolated from murine tumors using optimized enzymatic and density gradient protocols. Fresh tumor tissues were dissected, minced in digestion buffer (RPMI-1640 containing 0.5 mg/mL collagenase IV (Sigma, C5138) and 0.02 mg/mL DNase I (Sigma, 10104159001)), and incubated at 37 °C for 30–60 minutes with intermittent agitation. Digested suspensions were filtered (40 µm) and centrifuged (2,200 rpm, 7 min) to obtain single-cell mixtures. For TIL enrichment, cell pellets were resuspended in 30% Percoll, layered over a 70% Percoll gradient, and centrifuged (1,260g, 20 min). The intermediate lymphocyte-enriched layer was collected, washed, and pelleted (3,000 rpm, 10 min), yielding high-purity TILs.

### CircRNA vaccine preparations

Clean-PIE circularization elements and the P1A-Y37C protein coding region were chemically synthesized and cloned into a linearized pUC57 plasmid digested with a restriction enzyme. DNA synthesis and gene cloning were customized and performed by Suzhou Genewitz (Suzhou, China). CircRNA precursors were synthesized by *in vitro* transcription from a linearized plasmid DNA template using a Purescribe T7 High Yield RNA Synthesis Kit (CureMed, Suzhou, China). After the IVT reaction (*in vitro* transcription), DNA templates were digested by DNase I (CureMed, Suzhou, China) for 15 minutes. The linear RNA precursor was column-purified using a GeneJET RNA Purification Kit (Thermo Fisher Scientific, USA). For the generation of circRNA, the purified RNA precursor was incubated with guanosine triphosphate (GTP) with the concentration of 2 mM and a buffer containing magnesium (50 mM TrisHCl, [pH 8.0], 10 mM MgCl_2_, 1 mM DTT; Thermo Fisher Scientific). RNA was then heated for 15 min at 55 °C and purified through GeneJET RNA Purification Kit (Thermo Fisher Scientific, USA). The circularized RNA was purified by HPLC. RNA was loaded onto a 30*300 mm size exclusion column (SEC) with a particle size of 5 mm and a pore size of 1,000Å (Sepax Technologies, Suzhou, China) on an SCG (Sepure Instruments) protein purification system (Sepure Instruments, Suzhou, China). Then the column was eluted with RNase-free phosphate buffer (pH 6) and chromatography was performed at a flow rate of 15 mL/min. Chromatograms were recorded at a wavelength of 260 nm. RNA with high purity was collected by peak capture and concentrated, and the buffer was replaced with RNase-free water following ultracentrifugation. CircRNA-LNP complexes were generated through microfluidic devices (Micro&Nano Technologies, Shanghai, China). Purified circular mRNA was dissolved in citric acid buffer, and lipids (SM-102, Cholesterol, DSPC and DMG-PEG 2000) were dissolved in ethanol. The liquid flow rate was set as 12 mL/min, and mRNA/lipids (v/v) were used at a 3:1 ratio. CircRNA-LNP complexes were purified by filtration.

### Generation of dendritic cells

CD14^+^ cells were obtained by magnetic separation (BioLegend) from cryopreserved PBMCs (Shanghai Saily Biotechnology Co., Ltd., Cat. No. SLB-HP100B) to generate DCs. CD14^+^ cells were then cultured in complete RPMI-1640 medium containing 800 IU/mL granulocyte macrophage colony stimulating factor (PeproTech)and 500 IU/mL interleukin (IL)-4 (PeproTech). Cultures were fed with fresh medium and cytokines every 2–3 days. Mature DCs were generated by adding 10 ng/mL IL-1β, 10 ng/mL IL-6, 10 ng/mL tumor necrosis factor alpha, and 1 μg/mL PGE2 for 48 hours starting on day 5.

### Generation of peptide-specific T cells

Mature DCs were loaded with 20 μg/mL REISN peptides and 0.5 mM tris(2-carboxyethyl) phosphine at 37 °C for 2 hours. CD8^+^ naive T cells were enriched from autologous CD14^-^ cells by negative selection (Stemcell). Isolated CD8^+^ naive T cells were cocultured with peptide-loaded DCs in T cell medium (TexMACS with 10% human serum, 1% penicillin/streptomycin, 60 ng/mL IL-21, 10 IU/mL IL-2, 10 IU/mL IL-7, and 10 IU/mL IL-15). After 7 days, T cells were restimulated with peptide-loaded DCs. After 14 days, T cells were stained with PE-tetramer and FITC-CD8 and analyzed by flow cytometry.

### Generation of TCR041-T cells

PBMCs (Shanghai Saily Biotechnology Co., Ltd., Cat. No. SLB-HP100B) were thawed, resuspended in RPMI-1640 medium supplemented with 10% heat-inactivated fetal bovine serum (FBS; AU grade) and 1% penicillin-streptomycin, and activated at 1 × 10^6^ cells/mL with TransAct™ human T cell activation beads (Miltenyi Biotec, 10 μL/mL) in 24-well plates. On day 2, cells were transduced twice with lentiviral vectors encoding TCR041 (MOI = 1 per dose) in the presence of protamine sulfate (1:100 dilution). TCR expression was assessed on day 5 via flow cytometry using APC-anti-mouse TCRβ (clone H57-597) and FITC-anti-human CD8 antibodies (clones OKT4/SK1; BioLegend), with viability >90% confirmed by Trypan Blue exclusion. Transduced T cells were expanded in medium containing 200 IU/mL IL-2 (PeproTech), maintained at 5×10^5^ – 8×10^5^ cells/mL, and cryopreserved at 2×10^7^ cells/mL.

### *In vivo* mouse models

The animal experiments were approved by the Guangzhou Medical University Animal Ethics Committee (Reg. No.: GY2022-189). Female BALB/c mice (6–8 weeks) were purchased from Guangdong Yaokang Biotechnology Company. For tumorigenicity assays, CT26-P1A-Y37C cells (2×10^6^) and CT26-P1A-WT (2×10^6^) were injected subcutaneously into the flank of recipient BALB/c mice. CT26-arP1A (1×10^7^) and CT26-arNT (1×10^7^) were injected subcutaneously into BALB/c mice in a volume of 200 μL 50% Matrigel Basement Membrane Matrix (Corning) diluted in PBS. After transplantation, BALB/c mice were treated with 0.2% doxycycline (Dyets, DOX2000) once every two days. Tumor size was measured twice per week and volumes were calculated using the formula: volume (mm^3^) = length×width^2^/2. All mice were sacrificed at the experiment endpoint, and tumors were excised and weighed.

### Statistical analysis

Statistical analyses were performed using Prism V.9.5. Differences between two groups were evaluated using a two-tailed unpaired Student’s t-test, or paired t-tests for matched datasets where appropriate. For experiments involving two independent variables, statistical significance was assessed using two-way ANOVA, as appropriate for the experimental design, followed by the indicated *post hoc* multiple-comparisons test (for example, Tukey test).Tumor growth curves between treated and control groups were compared using two-way ANOVA, followed by Bonferroni *post hoc* tests for multiple comparisons. Data are presented as mean ± SD. *P* values < 0.05 were considered statistically significant.

## Results

### Rational design of REISN epitopes targeting TCR contact residues on MHC-I- presented peptides of TAA

Given the compounding factors affecting epitope immunogenicity, including gene expression patterns, RNA splicing, proteasomal processing, and MHC peptide loading/presentation, we prioritized clinically validated HLA-A*02:01-restricted TAA epitopes with confirmed MHC presentation for REISN engineering (20, 38). Employing this same design principle, we engineered REISN-targetable epitopes, including: AFP (FMNKFIYEI to FMNRFIYEI or FMNKFVYEI), MAGE-A1 (KVLEYVIKV to KVLECVIKV), MAGE-A10 (GLYDGMEHL to GLCDGMEHL), PRAME (SLLQHLIGL to SLLQHLVGL) and SSX2 (KASEKIFYV to KASERVFYV) (39, 40) (**Table 1**). NetMHCPan-4.1 analysis confirmed that all engineered variants maintain HLA-A*02:01 binding affinities <50 nM (**Table 1**). This systematic expansion demonstrates REISN’s versatility in targeting conserved human tumor antigens across diverse cancer types.

**Table 1:** Summary of REISN design for TAA editing targeting TCR interface region.

TAA	Species	WT peptide	Affinity (nM)	REISN peptide*	MHC	REISN Affinity (nM)	Target mRNA**	MS2-arRNA***
P1A	Mouse	LPYLGWLVF (35-43)	32.82	LPCLGWLVF	H-2L^d^	76.44	CUGCCUUAUCUAGGGUGGCUGGUCUUC	CTAGACAAGGCAGAATTTCTT
AFP	Human	FMNKFIYEI (158-166)	2.2	FMNKFVYEI	HLA-A*02:01	2.3	UUCAUGAACAAAUUCAUUUAUGAGAUA	ATAAACGAATTTGTTCATGAA
AFP	Human	FMNKFIYEI (158-166)	2.2	FMNRFIYEI	HLA-A*02:01	2.2	UUCAUGAACAAAUUCAUUUAUGAGAUA	TGAATCTGTTCATGAATGTCT
MAGEA1	Human	KVLEYVIKV (278-286)	5.83	KVLECVIKV	HLA-A*02:01	12.69	AAAGUCCUUGAGUAUGUGAUCAAGGUC	TCACACACTCAAGGACTTTCA
MAGEA10	Human	GLYDGMEHL (254-262)	6.96	GLCDGMEHL	HLA-A*02:01	51.4	GGGCUGUAUGAUGGGAUGGAGCACCUC	CATCACACAGCCCCATCATAT
PRAME	Human	SLLQHLIGL (425-433)	8.82	SLLQHLVGL	HLA-A*02:01	9.55	AGUCUCCUGCAGCACCUCAUCGGGCUG	CCCGACGAGGTGCTGCAGGAG
SSX2	Human	KASEKIFYV (41-49)	15.3	KASERVFYV	HLA-A*02:01	21.77	AAAGCCUCGGAGAAAAUCUUCUAUGUG	AGATTCTCTCCGAGGCTTTCA

* The target TCR interface amino acids are highlighted in red.

** The targeted adenines in A-to-I RNA editing are highlighted in red.

*** The MS2 sequence (aacatgaggatcacccatgtc) is appended to both termini of the arRNA for cloning. The cytidines mispairing with the targeted adenosines are highlighted in red.

To validate the REISN strategy in mice, we selected P1A, a well-characterized murine Melanoma Antigen Gene (MAGE)-type antigen, as a model system. The immunodominant epitope P1A_35-43_ (LPYLGWLVF) is efficiently presented by H-2L^d^ MHC class I molecules, with a predicted binding affinity of 33 nM as determined by NetMHCPan-4.1 algorithmic analysis (**Table 1**). Through rational design, we engineered P1A-REISN to introduce a site-specific A-to-I RNA editing event at codon 37, resulting in a tyrosine-to-cysteine substitution (Y37C). In canonical 9-mer epitopes, positions 2 and 9 serve as primary anchor residues for H-2L^d^ binding (41). The Y37C substitution strategically modifies the TCR interaction interface while preserving these critical MHC-I anchoring motifs. Computational modeling using NetMHCPan-4.1 predicts that this engineered neoepitope (LPCLGWLVF) retains substantial MHC binding capacity with an estimated affinity of 76 nM, a 2.3-fold reduction compared to the wild-type epitope, yet well within the threshold for immunogenic epitope presentation (typically <500 nM).

**Figure 1:**
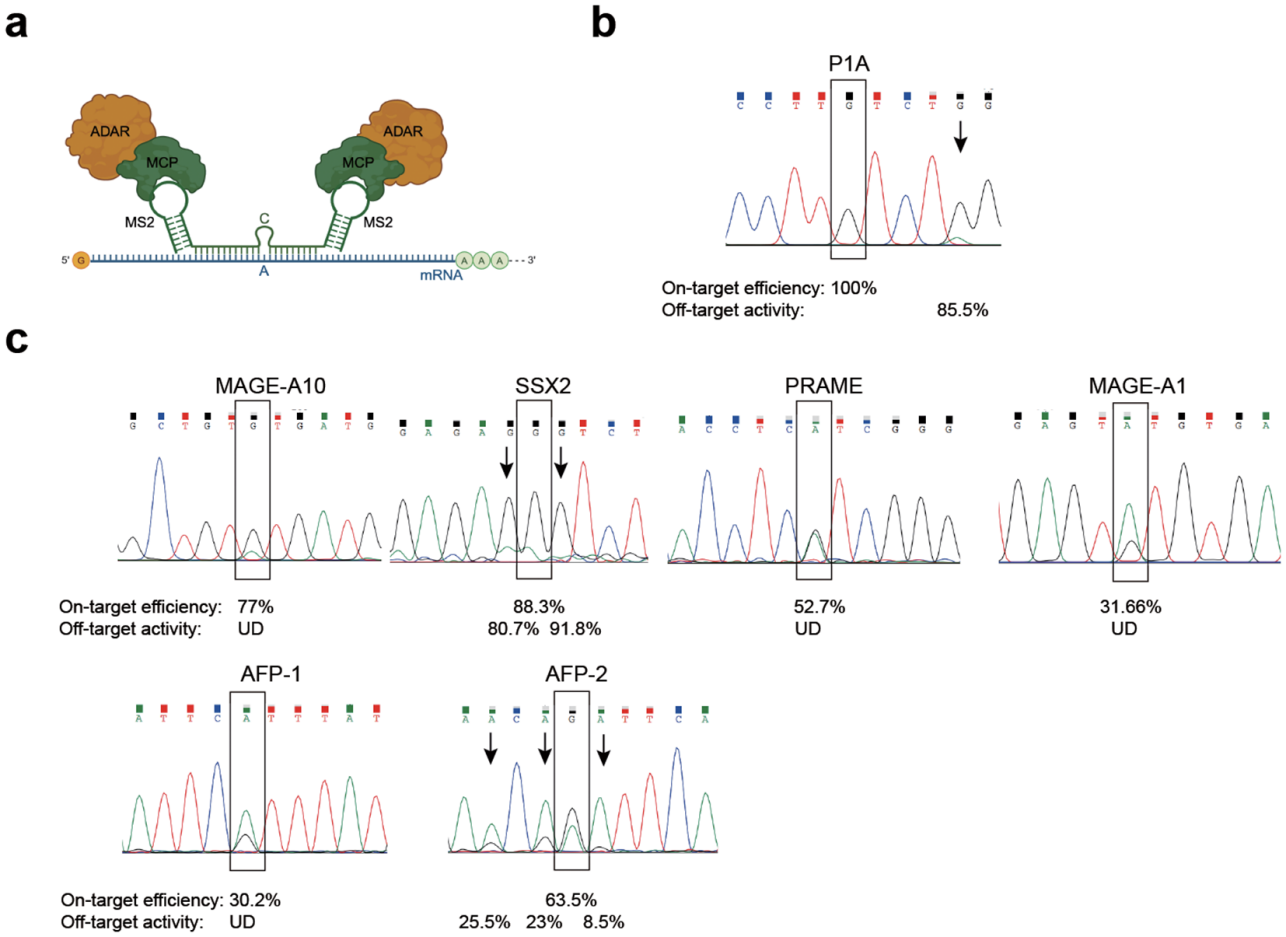
MCP-ADAR system engineers REISN epitopes at TCR interaction surfaces. **(a)** Schematic of the MCP-ADAR RNA editing platform. The engineered ADAR deaminase, fused to MCP, specifically binds MS2-arRNA via MCP-MS2 interactions. **(b)** Editing efficiency validation at murine target site P1A. Sequence chromatogram of P1A transcripts in Hela cells transiently transfected with plasmids encoding hADAR1, P1A, and arP1A. Data are from one of three independent experiments. **(c)** Editing efficiency validation at human target sites. Representative Sanger sequencing chromatograms are shown for *MAGE-A10*, *SSX2*, *PRAME*, *MAGE-A1*, and *AFP* in HeLa cells transiently transfected with plasmids encoding MCP-ADAR, target gene and corresponding arRNAs. The black boxes highlight on-target editing sites, while black arrows indicate bystander sites, with corresponding editing efficiencies labeled. Data are representative of two independent experiments. Image created with BioRender.com with permission. NT, non-target; UD, undetectable.

To enable precise editing of the selected TAA epitopes, we designed ADAR-recruiting RNAs (arRNAs) based on the MS2-MCP RNA editing system, as previously described by Katrekar et al. (29). Each gRNA contains a 21-nt antisense sequence complementary to the target TAA mRNA and two MS2 stem-loops at the 5’ and 3’ ends for MCP-ADAR fusion protein recruitment (**Figure 1a**). HeLa cells were co-transfected with plasmids encoding target cDNA, MCP-ADAR1, and MS2-arRNA; editing efficiency was quantified via RT-PCR and Sanger sequencing at 48 hours post-transfection. This approach achieved 30.2-80.7% editing efficiencies across target sites in both murine and human systems (**Figures 1b, c**). While efficient on-target editing was achieved, off-target activity was observed in 3 out of 7 REISNs we designed. At the P1A locus (100% in HeLa cells), we observed significant off-target activity at a 3’-adjacent TAG site (position + 4 relative to target, 85.5%), resulting in a synonymous mutation, that did not compromise the intended immunogenicity of the REISN-engineered neoepitope (**Figure 1b**). Interestingly, in the SSX2 model, editing (88.3%) spread to flanking adenosines (positions -1 and +1 relative to target, 80.7% and 91.8%, respectively), converting KASEKIFYV → KASERVFYV (two amino acid changes). In the AFP-2 REISN model, editing (63.5%) spread to flanking adenosines (positions -3, -1and +1 relative to target, 25.5%, 23% and 8.5%, respectively), converting FMNKFIYEI → FMSGFIYEI (two amino acid changes) and serendipitously creating an immunogenic variant (**Figure 1c**).

### Immunogenicity of REISN-engineered TCR contact mutations in murine models

**Figure 2:**
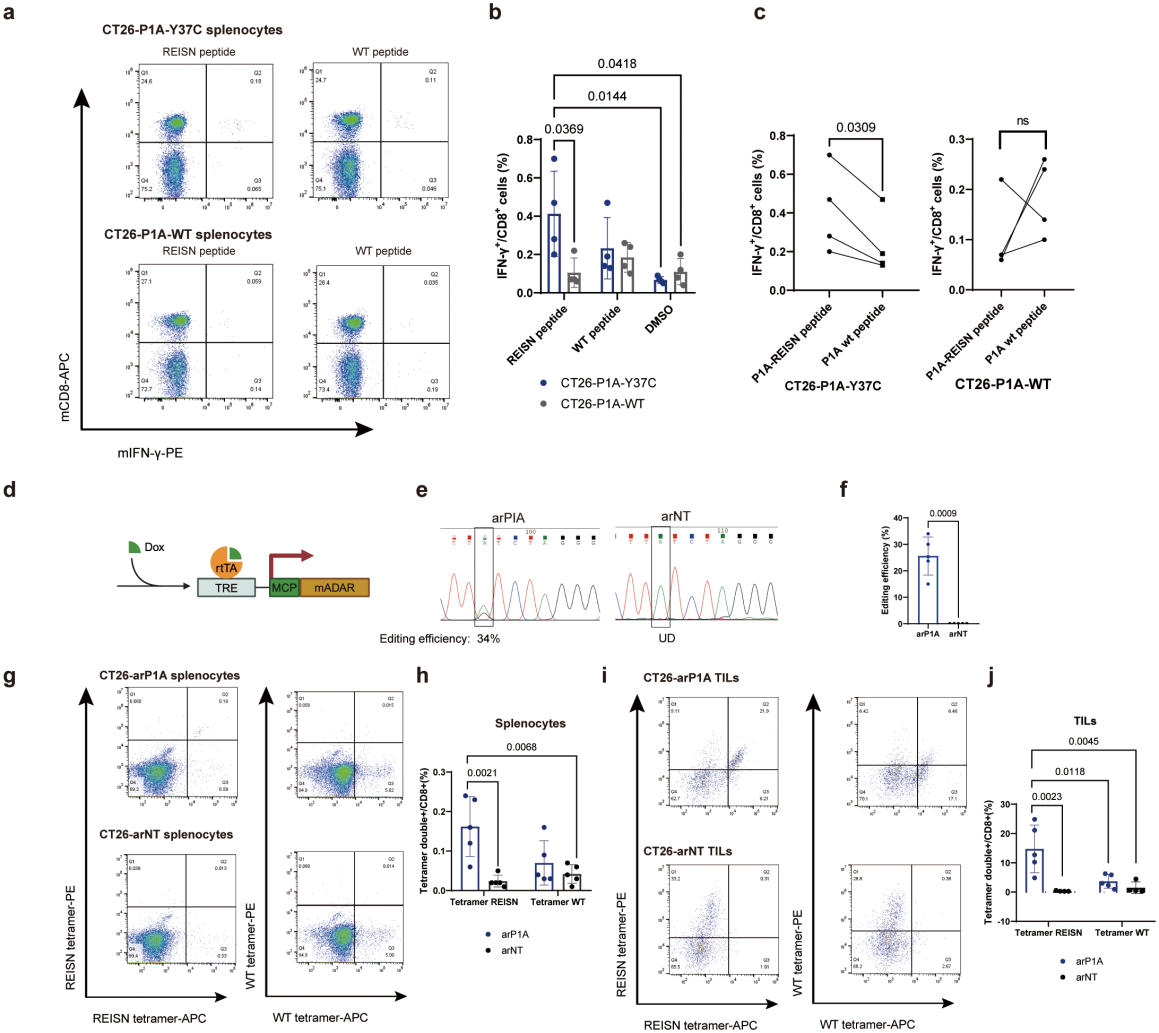
REISN-engineered tumors prime neoantigen-specific T cells. **(a-c)**, Intracellular cytokine staining (ICS) of splenic CD8^+^ T cells from BALB/c mice inoculated with CT26-P1A-Y37C or CT26-P1A-WT tumors. Splenocytes from mice bearing CT26-P1A-Y37C (*n* = 4) or CT26-P1A-WT (*n* = 4) tumors were stimulated for 12 hours with P1A-REISN peptide, P1A-WT peptide, or DMSO (negative control), followed by flow cytometry analysis. Shown are representative flow cytometry plots **(a)**, quantification of IFN-γ^+^ CD8^+^ T cells **(b)**, and paired comparison of T cell responses to P1A-REISN versus P1A-WT peptides **(c)**, Data are from one representative experiment with four mice for each group (n = 4). **d**, Schematic representation of the Dox-inducible RNA editing system. arRNA-MS2 is constitutively expressed (U6 promoter), while MCP-ADAR is under a tetracycline-responsive element (TRE) promoter. Image created with BioRender.com with permission. **(e, f)**, Representative chromatograms **(e)** and quantification **(f)** of editing efficiency in CT26-arP1A (left) versus CT26-arNT (right) groups. CT26 cells carrying the indicated editing components were implanted subcutaneously (2 × 10^^6^ cells per mouse), and mice were maintained on doxycycline chow (0.2% doxycycline) for more than 14 days to induce RNA editing *in vivo* before tumor collection and RT–PCR sequencing. Data are from one experiment with five mice for each group (n = 5). **(g, h)**, Representative tetramer staining **(g)** and quantification **(h)** of splenic CD8^+^ T cells from BALB/c mice inoculated with CT26-arP1A or CT26-arNT tumors. Data are from one representative experiment (*n* = 5 per group). **(i**, **j)**, Representative tetramer staining of tumor-infiltrating lymphocytes (TILs) **(i)** and quantification **(j)** of antigen-specific CD8^+^ TILs in CT26-arP1A versus CT26-arNT tumors. Data are represented as mean ± SD. Statistical significance in **(b)**, **(h)** and **(j)** was determined by two-way ANOVA, followed by Tukey’s *post hoc* multiple-comparisons test. Statistical significance in **(c)** was determined by paired two-tailed t-test. Statistical significance in **(f)** was determined by unpaired two-tailed Student’s t-test.

We then pursued proof-of-concept *in vivo* targeting P1A-REISN in immunocompetent BALB/c mice with H-2L^d^ background for epitope presentation. To evaluate the immunogenicity of the P1A-REISN epitope, we first generated isogenic cell lines stably expressing either wild-type P1A (CT26-P1A-WT) or the Y37C mutant (CT26-P1A-Y37C) through lentiviral transduction. BALB/c mice were subcutaneously inoculated with 2×10^6^ tumor cells. Three weeks post-inoculation, splenocytes were harvested and subjected to ex vivo stimulation with 10 μM of either the P1A-REISN peptide (LPCLGWLVF) or wild-type peptide (LPYLGWLVF) for 18 hours. Antigen-specific T cell responses were quantified via intracellular cytokine staining (ICS) for IFN-γ^+^CD8^+^ T cells (**Figure 2a**). Mice bearing CT26-P1A-Y37C tumors exhibited a statistically significant increase in IFN-γ^+^CD8^+^ T cells (0.413 ± 0.223%) upon REISN peptide stimulation compared to wild-type peptide (0.105 ± 0.077%, *p* = 0.037, two-way ANOVA), demonstrating neoepitope-specific priming (**Figures 2b, c**). In contrast, CT26-P1A-WT tumor-bearing mice showed no significant difference in IFN-γ responses to either peptide, consistent with central deletion of high-avidity T cell clones against the native epitope (**Figure 2c**). This orthogonal validation establishes that mutating the TCR interface amino acid generates *de novo* immunogenic epitopes capable of bypassing central tolerance.

Next, we engineered parallel CT26-arP1A and CT26-arNT cell lines to achieve stable *in vivo* P1A-RNA editing. To circumvent immune recognition of human ADAR in murine models, we substituted the human enzyme with its murine ortholog and cloned it downstream of a tetracycline-responsive element (TRE) with *pCW57* backbone, enabling doxycycline (Dox)-dependent expression (**Figure 2d**). Expression of P1A and MCP-ADAR was achieved by lentiviral mediated transduction at a multiplicity of infection (MOI) of 2 in CT26 murine colon carcinoma cells, which lack endogenous P1A expression, followed by transduction and selection of arP1A or arAFP lentivirus at a MOI of 10 (CT26-arP1A or CT26-arNT). Mice inoculated with CT26-arP1A and fed with Dox chow (0.2% doxycycline) showed sustained RNA editing (25.6 ± 7.2%) over 14 days at the P1A-REISN locus by Sanger sequencing, whereas in CT26-arNT (arAFP) controls, editing was undetectable (**Figures 2e, f**). As such, we engineered CT26-arP1A and CT26-arNT stable cell lines to achieve robust *in vivo* P1A-REISN induction.

We then established parallel challenge models by subcutaneously inoculating BALB/c mice with CT26-arP1A and CT26-arNT. To minimize background, two H-2L^d^/P1A-REISN tetramer -PE and tetramer-APC were stained simultaneously. Tetramer staining of splenocytes at day 15 revealed a 2.3-fold increase in antigen-specific CD8^+^ T cells in CT26-arP1A-bearing mice compared to CT26-arNT controls (0.162 ± 0.076% vs. 0.070 ± 0.056% of CD8^+^ T cells, *p* = 0.0021, two-way ANOVA, **Supplementary Figure 1** and **Figures 2g, h**). Flow cytometry analysis of dissociated tumors demonstrated striking enrichment of REISN-specific tumor-infiltrating lymphocytes (TILs) in CT26-arP1A mice (14.756 ± 8.136% of CD8^+^ T cells) versus minimal background in controls (0.363 ± 0.153%, *p* < 0.0001, **Supplementary Figure 1** and **Figures 2i, j**). The significantly higher REISN-specific TIL frequency (tumor/spleen ratio: 91) suggests robust recruitment and expansion of antigen-specific T cells within the tumor microenvironment. These results confirm that REISN-engineered tumors break immune tolerance and sustain neoantigen-specific T cell responses both systemically and intratumorally.

### Vaccination with REISN neoantigens generates specific antitumor immunity

**Figure 3:**
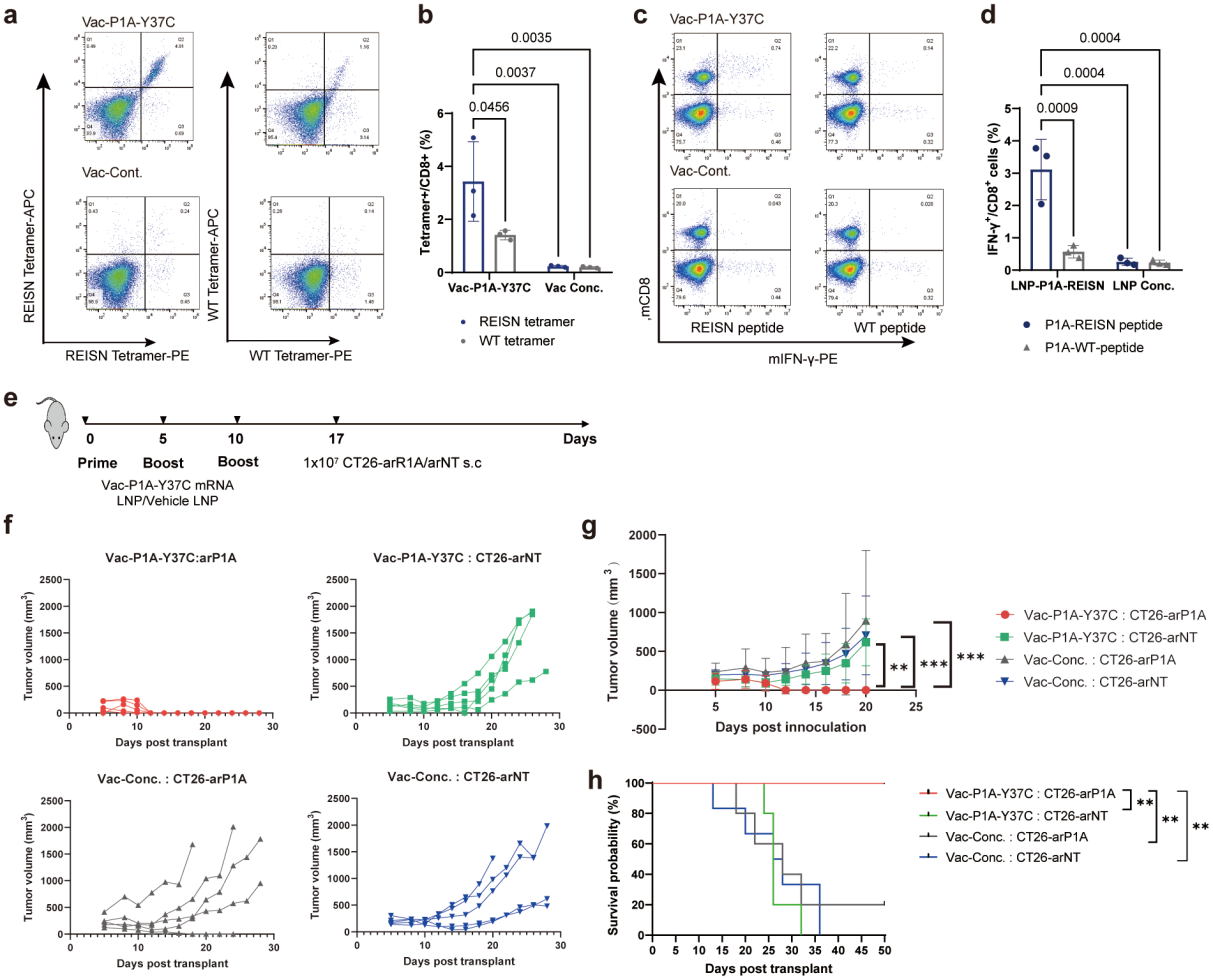
Vaccination with REISN neoantigens generates specific antitumor immunity. **(a-d)**, The immunogenic potency of the Vac-P1A-Y37C circRNA vaccine. Female BALB/c mice were treated with Vac-P1A-Y37C LNP (30 μg/mouse) or Vac-Conc. (30 μg/mouse, *n* = 3 per group) by intramuscular injection three times every five days. Splenocytes were harvested and analyzed by flow cytometry at day 17. Representative flow cytometry plots **(a)** and quantification **(b)** of REISN-tetramer^+^ or WT-tetramer^+^ (PE/APC double-positive) CD8^+^ T cells are shown. For intracellular cytokine staining (ICS), splenocytes from each group stimulated with P1A-REISN peptide or P1A-WT peptide for 12 hours. Representative flow cytometry plots **(c)** and quantification **(d)** are shown. Data are presented as mean ± SD from one experiment with 3 mice per group (n = 3). **e-h**, Vaccination with REISN neoantigens inhibits tumor growth. **(e)** Schematic of the BALB/c mice immunization and tumor cell transplantation protocol. Female BALB/c mice were treated with Vac-P1A-Y37C circRNA vaccine (30 μg/mouse) or Vac-Conc. (30 μg/mouse) by intramuscular injection three times every five days. 1×10^7^ CT26-arP1A or 1×10^7^ CT26-arNT cells were injected subcutaneously (*n* = 5 mice per group). Tumor size was measured every two days. Growth curve of individual groups **(f)** and combined chart **(g)**, and Kaplan-Meier survival curve **(h)** are shown. Data are represented as mean ± SD. Statistical significance in **(b)**, **(d)** and **(g)** was determined by two-way ANOVA, followed by Tukey’s *post hoc* multiple-comparisons test.

While P1A-REISN expression in tumor cells induced REISN-specific T cell priming, CT26-P1A-Y37C tumors still progress without significant growth inhibition (**Supplementary Figure 2**). To amplify REISN-directed immunity, we developed a lipid nanoparticle (LNP)-formulated circular RNA (circRNA) vaccine encoding P1A-Y37C (Vac-P1A-Y37C). To assess the immunogenic potency of the Vac-P1A-Y37C, BALB/c mice received three intramuscular injections of LNP-formulated Vac-P1A-Y37C or Vac-Conc. (LNP only, 30 μg/dose) at 5-day intervals. Seven days post-final immunization, splenocytes were analyzed for REISN-specific T cell responses through tetramer staining and IFN-γ secretion to enumerate antigen-specific CD8^+^ T cells. Mice immunized with Vac-P1A-Y37C circRNA-LNP exhibited strong REISN-specific T cell expansion, with 3.430 ± 1.503% of CD8^+^ splenocytes being double positive for REISN tetramer, representing a 15-fold higher frequency compared to the Vac-Conc.-immunized group (0.227 ± 0.035%, *p* = 0.0037, two-way ANOVA) (**Figures 3a, b**). Splenocytes were stimulated ex vivo with 10 μM peptide for 18 hours followed by intracellular cytokine staining (ICS) (**Figures 3c, d**). Consistently, in Vac-P1A-Y37C group, REISN peptide stimulation induced IFN-γ^+^CD8^+^ T cell frequencies 12-fold greater than those observed in the Vac-Conc. group (3.113 ± 0.937% vs. 0.257 ± 0.110%, *p* = 0.0004) (**Figure 3d**). Compared to WT peptide stimulation, REISN peptide elicited a 5.5-fold greater response (3.113 ± 0.937% vs. 0.556 ± 0.196%, *p* = 0.0009), demonstrating epitope-specific functional priming (**Supplementary Figure 3**). The observed cross-reactivity against wild-type P1A suggests that REISN vaccination may provide dual protection against both edited and unedited tumor cell populations (**Figures 3b, d**).

Immunized mice were subcutaneously inoculated with 1×10^7^ CT26-arP1A or CT26-arNT cells (**Figure 3e**). Dox chow (200 mg/kg) was administered both groups to induce P1A-REISN expression. We observed that Vac-P1A-Y37C vaccination completely inhibited CT26-arP1A tumor growth compared to LNP controls (**Figures 3f-h**). No significant growth inhibition was observed against CT26-arNT tumors, confirming REISN-dependent antitumor immunity (**Figures 3f, g**). These results establish that circRNA vaccines targeting P1A-REISN elicit potent CD8^+^ T cell responses capable of suppressing tumors with RNA-edited antigens in immunocompetent hosts.

### REISN activate antigen-specific naïve human T cells

**Figure 4:**
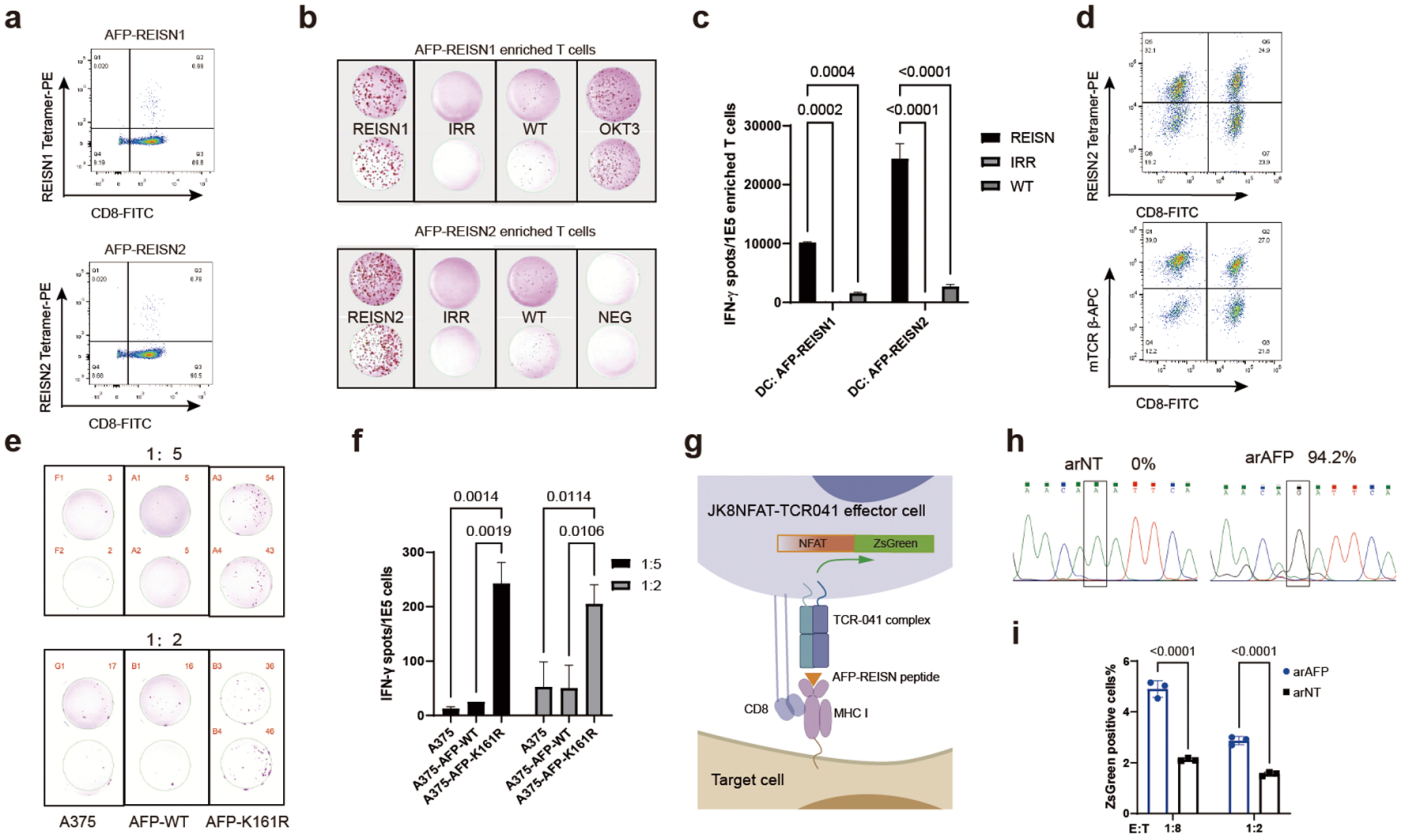
REISN activate antigen-specific naïve human T cells. **(A)** Stimulation and enrichment of healthy human peripheral blood-derived naïve T cells to obtain AFP-REISN-specific T cells. Naïve CD8^+^ T cells were isolated from two HLA-A*02:01-positive healthy donors and stimulated with dendritic cells (DCs) loaded with AFP-REISN1 or AFP-REISN2 peptides. After approximately 14 days in culture, stimulated T cells were stained with corresponding tetramers. Shown are representative tetramer staining flow plots. **(b, c)** Recognition of T2 cells pulsed with AFP-REISN peptides by enriched REISN-specific polyclonal T cells from two individual donors. Representative plots **(b)** and quantification **(c)** of ELISpot assay detecting IFN-γ secretion by AFP-REISN-specific T cells co-cultured with T2 cells pulsed with 10–^6^ M of the corresponding peptides. OKT3 antibody served as positive control; irrelevant peptide (IRR) (EBV: SSCSSCPLSK) was negative control. **(d)**, Representative flow cytometry plots of PBMCs transduced with lentiviral vectors expressing TCR041. **(e, f)**, Recognition of A375 melanoma cells stably expressing AFP-K161R by TCR041-T cells. Representative plots **(e)** and quantification **(f)** of ELISpot assay detecting IFN-γ secretion by TCR041-engineered T cells co-cultured with A375-AFP-K161R or A375-AFP-WT (effector: target ratios = 1:5 and 1:2). **g**, Schematic of JK8NFAT-TCR041 reporter system. Image created with BioRender.com with permission. **h**, RNA editing levels of AFP in 293T cells transiently transfected with plasmids encoding AFP-WT, MCP-ADAR1 and MS2-arAFP/arNT (representative chromatograms). **(i)**, ZsGreen expression in JK8NFAT-TCR041 reporter cells co-cultured with transfected 293T cells. Data are represented as mean ± SD. Statistical significance in **(c)**, **(f)** and **(i)** was determined by two-way ANOVA.

To evaluate the clinical potential of REISN, we established a T cell priming system targeting REISN designed for AFP, a clinically relevant oncofetal antigen (**Table 1**). We primed naïve CD8^+^ T cells from two HLA-A*02:01-positive, healthy donors with dendritic cells (DCs) loaded with AFP-REISN peptides (REISN1: FMNKFVYEI or REISN2: FMNRFIYEI) (**Table 1**). After 14 days of peptide stimulation, we could easily detect AFP-REISN1- and REISN2-specific CD8^+^ T cells by tetramer staining (**Figure 4a**). We then isolated AFP-REISN-specific T cells via CD8^+^/AFP-REISN tetramer sorting (purity >90%). To assess the function of these cells, we established co-culture with peptide-pulsed T2 cells. ELISpot assays demonstrated robust IFN-γ secretion specific to the corresponding AFP-REISN peptides (REISN donor1: 10,175 ± 106 vs. irrelevant controls: 25 ± 35 vs. WT: 1,525 ± 177 spots/10^5^ cells; REISN donor2: 24,375 ± 2581 vs. irrelevant controls: 0 ± 0 vs. WT: 2,675 ± 389 spots/10^5^ cells) (**Figures 4b, c**). The representative data shown are consistent across both donors, indicating that mutating TCR contact residue generates immunogenic neoepitopes recognizable by human T cells. This result also suggested that REISN strategy activate REISN-specific naïve T cells.

To facilitate the evaluation of the immunogenicity of AFP-REISN, we isolated single clones from AFP-REISN2-enriched T cell populations and identified a dominant TCR clone (TCR041) through single-cell sequencing. TCR alpha and beta chain pair in the β-α order was cloned into the lentiviral vector *pLKO.1* under EF1α promoter (**Supplementary Figure 4a**). The human TCR constant regions were replaced with murine TCR constant regions to ensure a preferred pairing of transgenic TCR chains. The murine constant regions were modified with a second disulfide bond and the LVL hydrophobic mutations in the TCRα transmembrane region (37). To characterize its specificity, we transduced healthy donor-derived PBMCs with lentiviral vectors expressing TCR041, followed by CD3/CD28 bead stimulation and expansion. Flow cytometry using REISN2 tetramer and mouse constant region antibody confirmed TCR041 surface expression in >50% of CD8^+^ T cells (**Figure 4d**). Functional assessment via ELISpot demonstrated that TCR041-T cells specifically recognized AFP-REISN2-pulsed T2 cells in a dose-responsive manner (10–^5^ M: 215.5 ± 17.678 IFN-γ spots/2000 cells), with slight cross-reactivity against wild-type AFP peptide at the highest concentration, 10–^5^ M (36 ± 5.657 spots/2000 cells), while maintaining negligible reactivity to irrelevant peptide-pulsed T2 cells (**Supplementary Figure 4b**, c). This molecular-level validation confirms that REISN-induced single-amino acid substitutions generate structurally distinct neoepitopes.

To validate endogenous processing and MHC-I presentation of AFP-REISN2 epitopes, we cocultured TCR041-transduced T cells with A375 cells stably expressing AFP-K161R. A375 cells, which lack basal expression of AFP, were used as negative control. ELISpot assays demonstrated significantly enhanced IFN-γ secretion towards A375-AFP-K161R cells compared to A375-AFP-WT cells (IFN-γ: 242.5 ± 38.9 vs. 25 ± 0 spots/10^5^ cells, *p* = 0.0019 at E: T ratio 1:5), suggesting efficient MHC-I presentation of AFP-REISN2 epitope in a human melanoma line (**Figures 4e, f**).

We then asked whether TCR041 could recognize AFP-REISN2 induced by ADAR mediated RNA editing. We used a previously established JK8NF reporter cell line derived from J.RT3-T3.5 cells, engineered to express CD8 for TCR support and ZsGreen under the control of the nuclear factor of activated T cells (NFAT) promoter as a reporter for T cell activation (**Figure 4g**) (37). We then transduced this reporter cell line with lentivirus overexpressing TCR041 (**Supplementary Figure 5a**). 293T cells, which have a low level of HLA-A*02:01 expression, were co-transfected with plasmids encoding AFP-WT, MCP-ADAR1 and MS2-arAFP/arNT; editing efficiency was 94.2% quantified via RT-PCR and Sanger-sequencing 48 hours post-transfection (**Figure 4h**). JK8-NFAT-TCR041 cells were then cocultured with transfected 293T cells for 24 hours. Flow cytometry analysis demonstrated that TCR041-engineered JK8NFAT cells detected AFP-REISN2 epitopes generated *de novo* in 293T cells through transient RNA editing (E:T ratio at 1:8, ZsGreen^+^: 4.903 ± 0.329% vs. 2.120 ± 0.061% in controls, *p* < 0.0001) (**Supplementary Figure 5** and **Figure 4i**). These results provide direct evidence that single-residue substitutions at TCR contact interfaces (K to R) enable escape from central tolerance of AFP while maintaining MHC-I anchoring. RNA-edited neoantigens undergo natural antigen processing and presentation pathways. Taken together, our data established REISN as a novel strategy to reprogram tumor immunogenicity.

## Discussion

Using murine cancer testis antigen P1A and tumor-associated fetal mammalian glycoprotein AFP as models, this study introduces REISN as a proof-of-concept strategy for cancer immunotherapy. The REISN strategy represents a novel application of RNA editing for de novo neoantigen induction through site-directed recoding of tumor-associated antigens (TAAs). By leveraging programmable RNA base editing, REISN addresses two fundamental limitations of conventional cancer vaccine strategies (1): the stochastic nature of tumor mutational burden (TMB), and (2) the immune tolerance barrier against tumor-associated antigens (TAAs). The strategy’s potential advantages include site-specific, reversibility and scalability. The transient nature of RNA editing circumvents risks associated with permanent genomic alterations, as edited transcripts are inherently labile and subject to natural turnover. This temporal control minimizes off-target persistence and eliminates ethical concerns linked to germline editing technologies. Our data show that REISN-engineered neoepitopes elicit potent, antigen-specific T cell responses capable of controlling tumor growth in preclinical models.

Cancer/testis antigens (CTAs, e.g., NY-ESO-1, MAGE-A3) and oncofetal antigens (e.g., AFP, GPC3) represent attractive REISN targets due to their tumor-restricted expression and inherent immunogenicity (39). Our strategy’s potential to break immune tolerance against these self-antigens suggests new therapeutic avenues for further investigation in cancers with low TMB, such as hepatocellular carcinoma (HCC) and pancreatic ductal adenocarcinoma (PDAC).

The selection of endogenous ADAR over bacterial-derived editors (e.g., Cas13) as the catalytic core of REISN is underpinned by immunological and translational considerations. Bacterial proteins, including Cas13, frequently elicit host immune responses due to their foreign origin. Pre-existing anti-Cas13 antibodies have been detected in >60% of human sera, posing a significant risk of immune-mediated clearance and reduced therapeutic efficacy (42). In contrast, ADAR-based strategies leverage an endogenous human RNA-editing enzyme with lower immunogenicity risk, ensuring sustained editing activity in vivo (43). In addition, ADAR’s constitutive expression in mammalian cells may facilitate efficient recruitment to target transcripts without requiring exogenous enzyme delivery (33–35, 44, 45). This intrinsic compatibility minimizes the complexity of therapeutic formulations and enhances editing precision through natural substrate recognition mechanisms. Collectively, these attributes suggest ADAR as a promising enzymatic scaffold for REISN, balancing editing efficiency with low immunogenicity risk in proof-of-concept settings.

The primary challenge for clinical translation of the REISN strategy lies in achieving efficient in vivo delivery to tumor cells. REISN is broadly compatible with existing RNA editing platforms, including CRISPR-Cas13 systems, endogenous ADAR-recruiting approaches (e.g., LEAPER, RESTORE), and chemically engineered guide RNA-oligonucleotides—all of which have shown feasibility in correcting pathogenic mutations underlying monogenic disorder (32–35, 44, 46–48). Of note, the current study employed an engineered ADAR expression platform. Exploring chemically modified guide RNAs or other endogenous ADAR-recruiting approaches represents a key direction for future development. We also acknowledge that the in vivo data presented here are based on pre-engineered CT26 models with overexpressed P1A antigen. While appropriate for proof-of-concept studies, these models do not recapitulate endogenous tumor biology (34).

Although REISN primarily employs ADAR-mediated A-to-I editing, its modular design provides the flexibility to incorporate alternative RNA-editing modalities, such as APOBEC3A-driven C-to-U conversion (49). In addition, REISN can be adapted to generate neoantigens presented by both MHC class I and II molecules, thereby simultaneously activating CD8+ cytotoxic T cells and CD4+ helper T cells, a critical feature for sustaining durable antitumor immunity (50). Machine learning algorithms (e.g., NetMHCpan-4.1) could be explored to assist REISN design by predicting edited epitopes with enhanced immunogenicity.

The observed off-target RNA editing events in our study present both challenges and opportunities. While the 100% on-target editing efficiency at the P1A locus demonstrates REISN’s precision, the concomitant 85.5% proximal editing at a downstream TAG site (P1A model) and multi-site adenosine editing in SSX2 (converting KASEKIFYV→KASERVFYV) and AFP-2 (FMNKFIYEI → FMSGFIYEI) reveal inherent limitations of native ADAR activity. Importantly, the SSX2 case suggests that such bystander editing may not be uniformly detrimental: the dual amino acid change may enhance immunogenicity since the wild-type epitope is self-tolerated, suggesting that under carefully controlled conditions, selected bystander editing events could be explored to amplify neoantigen diversity. Emerging studies have established several principled approaches to enhance the specificity of arRNA-guided RNA editing, which could be adapted for REISN optimization (32, 34, 44, 51, 52). This bifaceted approach, minimizing deleterious off-target effects while strategically leveraging beneficial ones, warrants further investigation in future REISN studies. We also note that in some cases, REISN-specific T cells showed modest recognition of the wild-type peptide. While this could be interpreted as a potential advantage for targeting both edited and unedited tumor cells, it also raises safety considerations. Although the TAAs used in this study (AFP and P1A) have highly restricted expression patterns—AFP is not expressed in adult tissues and P1A is expressed only in the immune-privileged testis—future applications of the REISN strategy to other TAAs with broader expression patterns should carefully evaluate the potential risks of wild-type cross-reactivity and on-target/off-tumor toxicity.

In conclusion, REISN strategy represents a novel proof-of-concept framework for cancer immunotherapy. By combining site-directed RNA engineering with immunology, REISN suggests a potential avenue for further exploration in tumors with limited neoantigen availability.

## Data Availability

The datasets presented in this study can be found in online repositories. The names of the repository/repositories and accession number(s) can be found in the article/**Supplementary Material**.

## Supplementary material

Supplementary Figure 1 Representative gating strategy for splenocytes and tumor infiltrating lymphocytes. **(a)**, Gating strategy for mouse splenocytes. **(b)**, Gating strategy for transplanted mouse tumor-infiltrating lymphocytes.

Supplementary Figure 2 P1A-REISN is insufficient to control tumor progression. Female BALB/c mice were transplanted with 2×10^6^ CT26-P1A-Y37C or 1×10^7^ CT26-P1A cells subcutaneously (*n* = 3 per group). Tumor size was measured every two days. Tumor volume is shown. Data are represented as mean ± SD.

Supplementary Figure 3 Vac-P1A-Y37C circRNA vaccine generate REISN-specific T cell response. **(a, b)**, Quantitation of intracellular cytokine staining of splenocytes from Vac-P1A-Y37C circRNA vaccine **(a)** and control group **(b)** stimulated with P1A-REISN peptide and P1A-WT peptide for 12 hours. Paired comparison of T cell responses to P1A-REISN versus P1A-WT peptides were shown. Statistical significance was determined by paired t-test.

Supplementary Figure 4 TCR041 T cell receptor mediates specific recognition of the AFP-REISN neoantigen. **(a)**, Schematic of TCR041 lentiviral expression vector assembly. **(b)**, Representative ELISpot assay detecting IFN-γ secretion by TCR041-engineered T cells co-cultured with T2 cells pulsed with AFP-REISN or AFP-WT peptides (10 μg/mL, 24 hours). Negative control: T2 cells without peptide. Positive control: PMA and ionomycin. Spot-forming units (SFUs) reflect antigen-specific T cell activation. **(c)**, Quantification of IFN-γ^+^ SFUs (*n* = 2 biologically independent experiments). Data are represented as mean ± SD.

Supplementary Figure 5 JK8NFAT-TCR041 cells detect AFP-REISN2 epitopes generated de novo in 293T cells through transient RNA editing. Representative flow cytometry plots of ZsGreen expression levels in JK8NFAT-TCR041 effector cells only **(a)**, co-cultured with 293T cells transiently transfected with plasmids encoding AFP, MCP-ADAR1, and arNT **(b)** or arAFP **(c)**

## References

[1] Sharma P Allison JP . The future of immune checkpoint therapy. Science. (2015) 348:56–61. doi: 10.1126/science.aaa8172 25838373

[2] Lin MJ Svensson-Arvelund J Lubitz GS Marabelle A Melero I Brown BD . Cancer vaccines: the next immunotherapy frontier. Nat Cancer. (2022) 3:911–26. doi: 10.1038/s43018-022-00418-6 35999309

[3] Dreno B Thompson JF Smithers BM Santinami M Jouary T Gutzmer R . MAGE-A3 immunotherapeutic as adjuvant therapy for patients with resected, MAGE-A3-positive, stage III melanoma (DERMA): a double-blind, randomised, placebo-controlled, phase 3 trial. Lancet Oncol. (2018) 19:916–29. doi: 10.1016/s1470-2045(18)30254-7 29908991

[4] Gure AO Chua R Williamson B Gonen M Ferrera CA Gnjatic S . Cancer-Testis genes are coordinately expressed and are markers of poor outcome in non–small cell lung cancer. Clin Cancer Res. (2005) 11:8055–62. doi: 10.1158/1078-0432.ccr-05-1203 16299236

[5] Lu X Deng S Xu J Green BL Zhang H Cui G . Combination of AFP vaccine and immune checkpoint inhibitors slows hepatocellular carcinoma progression in preclinical models. J Clin Invest. (2023) 133(11):e163291. doi: 10.1172/jci163291 37040183 PMC10231990

[6] Leko V Rosenberg SA . Identifying and targeting human tumor antigens for T cell-based immunotherapy of solid tumors. Cancer Cell. (2020) 38:454–72. doi: 10.1016/j.ccell.2020.07.013 32822573 PMC7737225

[7] Holm JS Funt SA Borch A Munk KK Bjerregaard A-M Reading JL . Neoantigen-specific CD8 T cell responses in the peripheral blood following PD-L1 blockade might predict therapy outcome in metastatic urothelial carcinoma. Nat Commun. (2022) 13(1):1935. doi: 10.1038/s41467-022-29342-0 35410325 PMC9001725

[8] Oliveira G Stromhaug K Klaeger S Kula T Frederick DT Le PM . Phenotype, specificity and avidity of antitumour CD8(+) T cells in melanoma. Nature. (2021) 596:119–25. doi: 10.1038/s41586-021-03704-y 34290406 PMC9187974

[9] Puig-Saus C Sennino B Peng S Wang CL Pan Z Yuen B . Neoantigen-targeted CD8+ T cell responses with PD-1 blockade therapy. Nature. (2023) 615:697–704. doi: 10.1038/s41586-023-05787-1 36890230 PMC10441586

[10] McGranahan N Furness AJ Rosenthal R Ramskov S Lyngaa R Saini SK . Clonal neoantigens elicit T cell immunoreactivity and sensitivity to immune checkpoint blockade. Science. (2016) 351:1463–9. doi: 10.1126/science.aaf1490 26940869 PMC4984254

[11] Hilf N Kuttruff-Coqui S Frenzel K Bukur V Stevanovic S Gouttefangeas C . Actively personalized vaccination trial for newly diagnosed glioblastoma. Nature. (2019) 565:240–245. doi: 10.1038/s41586-018-0810-y 30568303

[12] Hu Z Leet DE Allesoe RL Oliveira G Li S Luoma AM . Personal neoantigen vaccines induce persistent memory T cell responses and epitope spreading in patients with melanoma. Nat Med. (2021) 27:515–25. doi: 10.1038/s41591-020-01206-4 33479501 PMC8273876

[13] Keskin DB Anandappa AJ Sun J Tirosh I Mathewson ND Li S . Neoantigen vaccine generates intratumoral T cell responses in phase Ib glioblastoma trial. Nature. (2019) 565:234–9. doi: 10.1038/s41586-018-0792-9 30568305 PMC6546179

[14] Ott PA Hu Z Keskin DB Shukla SA Sun J Bozym DJ . An immunogenic personal neoantigen vaccine for patients with melanoma. Nature. (2017) 547:217–21. doi: 10.1038/nature22991 28678778 PMC5577644

[15] Sahin U Derhovanessian E Miller M Kloke BP Simon P Lower M . Personalized RNA mutanome vaccines mobilize poly-specific therapeutic immunity against cancer. Nature. (2017) 547:222–6. doi: 10.1038/nature23003 28678784

[16] Rojas LA Sethna Z Soares KC Olcese C Pang N Patterson E . Personalized RNA neoantigen vaccines stimulate T cells in pancreatic cancer. Nature. (2023) 618:144–50. doi: 10.1038/s41586-023-06063-y 37165196 PMC10171177

[17] Weber JS Carlino MS Khattak A Meniawy T Ansstas G Taylor MH . Individualised neoantigen therapy mRNA-4157 (V940) plus pembrolizumab versus pembrolizumab monotherapy in resected melanoma (KEYNOTE-942): a randomised, phase 2b study. Lancet. (2024) 403:632–44. doi: 10.1016/s0140-6736(23)02268-7 38246194

[18] Melief CJM . Cancer: Precision T-cell therapy targets tumours. Nature. (2017) 547:165–7. doi: 10.1038/nature23093 28678783

[19] Tran E Robbins PF Rosenberg SA . 'Final common pathway' of human cancer immunotherapy: targeting random somatic mutations. Nat Immunol. (2017) 18:255–62. doi: 10.1038/ni.3682 28198830 PMC6295671

[20] Blass E Ott PA . Advances in the development of personalized neoantigen-based therapeutic cancer vaccines. Nat Rev Clin Oncol. (2021) 18:215–29. doi: 10.1038/s41571-020-00460-2 33473220 PMC7816749

[21] Luksza M Sethna ZM Rojas LA Lihm J Bravi B Elhanati Y . Neoantigen quality predicts immunoediting in survivors of pancreatic cancer. Nature. (2022) 606:389–95. doi: 10.1038/s41586-022-04735-9 PMC9177421 35589842

[22] Roth SH Levanon EY Eisenberg E . Genome-wide quantification of ADAR adenosine-to-inosine RNA editing activity. Nat Methods. (2019) 16:1131–8. doi: 10.1038/s41592-019-0610-9 31636457

[23] Ramaswami G Lin W Piskol R Tan MH Davis C Li JB . Accurate identification of human Alu and non-Alu RNA editing sites. Nat Methods. (2012) 9:579–81. doi: 10.1038/nmeth.1982 22484847 PMC3662811

[24] Quin J Sedmik J Vukic D Khan A Keegan LP O'Connell MA . ADAR RNA modifications, the epitranscriptome and innate immunity. Trends Biochem Sci. (2021) 46:758–71. doi: 10.1016/j.tibs.2021.02.002 33736931

[25] Booth BJ Nourreddine S Katrekar D Savva Y Bose D Long TJ . RNA editing: Expanding the potential of RNA therapeutics. Mol Ther J Am Soc Gene Ther. (2023) 31:1533–49. doi: 10.1016/j.ymthe.2023.01.005 36620962 PMC9824937

[26] Pfeiffer LS Stafforst T . Precision RNA base editing with engineered and endogenous effectors. Nat Biotechnol. (2023) 41:1526–42. doi: 10.1038/s41587-023-01927-0 37735261

[27] Sheridan C . Shoot the messenger: RNA editing is here. Nat Biotechnol. (2023) 41:306–8. doi: 10.1038/s41587-023-01709-8 36879010

[28] Vogel P Moschref M Li Q Merkle T Selvasaravanan KD Li JB . Efficient and precise editing of endogenous transcripts with SNAP-tagged ADARs. Nat Methods. (2018) 15:535–8. doi: 10.1038/s41592-018-0017-z 29967493 PMC6322650

[29] Katrekar D Chen G Meluzzi D Ganesh A Worlikar A Shih YR . *In vivo* RNA editing of point mutations via RNA-guided adenosine deaminases. Nat Methods. (2019) 16:239–42. doi: 10.1038/s41592-019-0323-0 30737497 PMC6395520

[30] Merkle T Merz S Reautschnig P Blaha A Li Q Vogel P . Precise RNA editing by recruiting endogenous ADARs with antisense oligonucleotides. Nat Biotechnol. (2019) 37:133–8. doi: 10.1038/s41587-019-0013-6 30692694

[31] Qu L Yi Z Zhu S Wang C Cao Z Zhou Z . Programmable RNA editing by recruiting endogenous ADAR using engineered RNAs. Nat Biotechnol. (2019) 37:1059–69. doi: 10.1038/s41587-019-0178-z 31308540

[32] Katrekar D Yen J Xiang Y Saha A Meluzzi D Savva Y . Efficient *in vitro* and *in vivo* RNA editing via recruitment of endogenous ADARs using circular guide RNAs. Nat Biotechnol. (2022) 40:938–45. doi: 10.1038/s41587-021-01171-4 35145312 PMC9232839

[33] Monian P Shivalila C Lu G Shimizu M Boulay D Bussow K . Endogenous ADAR-mediated RNA editing in non-human primates using stereopure chemically modified oligonucleotides. Nat Biotechnol. (2022) 40:1093–102. doi: 10.1038/s41587-022-01225-1 35256816

[34] Reautschnig P Wahn N Wettengel J Schulz AE Latifi N Vogel P . CLUSTER guide RNAs enable precise and efficient RNA editing with endogenous ADAR enzymes *in vivo*. Nat Biotechnol. (2022) 40:759–768. doi: 10.1038/s41587-021-01105-0 34980913

[35] Yi Z Qu L Tang H Liu Z Liu Y Tian F . Engineered circular ADAR-recruiting RNAs increase the efficiency and fidelity of RNA editing *in vitro* and *in vivo*. Nat Biotechnol. (2022) 40:946–55. doi: 10.1038/s41587-021-01180-3 35145313

[36] Reautschnig P Fruhner C Wahn N Wiegand CP Kragness S Yung JF . Precise *in vivo* RNA base editing with a wobble-enhanced circular CLUSTER guide RNA. Nat Biotechnol. (2025) 43:545–57. doi: 10.1038/s41587-024-02313-0 38997581 PMC11994451

[37] Xiong C Huang L Kou H Wang C Zeng X Sun H . Identification of novel HLA-A*11:01-restricted HPV16 E6/E7 epitopes and T-cell receptors for HPV-related cancer immunotherapy. J Immunother Cancer. (2022) 10:e004790. doi: 10.1136/jitc-2022-004790 36180070 PMC9528665

[38] Dersh D Hollý J Yewdell JW . A few good peptides: MHC class I-based cancer immunosurveillance and immunoevasion. Nat Rev Immunol. (2020) 21:116–28. doi: 10.1038/s41577-020-0390-6 32820267

[39] Baulu E Gardet C Chuvin N Depil S . TCR-engineered T cell therapy in solid tumors: State of the art and perspectives. Sci Adv. (2023) 9:eadf3700. doi: 10.1126/sciadv.adf3700 36791198 PMC9931212

[40] Golikova EA Alshevskaya AA Alrhmoun S Sivitskaya NA Sennikov SV . TCR-T cell therapy: current development approaches, preclinical evaluation, and perspectives on regulatory challenges. J Transl Med. (2024) 22:897. doi: 10.1186/s12967-024-05703-9 39367419 PMC11451006

[41] Tadros DM Eggenschwiler S Racle J Gfeller D . The MHC Motif Atlas: a database of MHC binding specificities and ligands. Nucleic Acids Res. (2023) 51:D428–d37. doi: 10.1093/nar/gkac965 36318236 PMC9825574

[42] Charlesworth CT Deshpande PS Dever DP Camarena J Lemgart VT Cromer MK . Identification of preexisting adaptive immunity to Cas9 proteins in humans. Nat Med. (2019) 25:249–54. doi: 10.1038/s41591-018-0326-x 30692695 PMC7199589

[43] Liao Y Jung SH Kim T . A-to-I RNA editing as a tuner of noncoding RNAs in cancer. Cancer Lett. (2020) 494:88–93. doi: 10.1016/j.canlet.2020.08.004 32822814

[44] Jauregui-Matos V Jacobs O Ouye R Mozumder S Salvador Prince J Fink Kyle D . Site-specific regulation of RNA editing with ribose-modified nucleoside analogs in ADAR guide strands. Nucleic Acids Res. (2024) 52:6733–47. doi: 10.1093/nar/gkae461 38828787 PMC11229365

[45] Zhang Y Feng D Mu G Wang Q Wang J Luo Y . Light-triggered site-directed RNA editing by endogenous ADAR1 with photolabile guide RNA. Cell Chem Biol. (2023) 30:672–82.e5. doi: 10.1016/j.chembiol.2023.05.006 37295425

[46] Nose K Hidaka K Yano T Tomita Y Fukuda M . Short-chain guide RNA for site-directed A-to-I RNA editing. Nucleic Acid Ther. (2021) 31:58–67. doi: 10.1089/nat.2020.0866 33170095

[47] Yi Z Zhao Y Yi Z Zhang Y Tang G Zhang X . Utilizing AAV-mediated LEAPER 2.0 for programmable RNA editing in non-human primates and nonsense mutation correction in humanized Hurler syndrome mice. Genome Biol. (2023) 24:243. doi: 10.1186/s13059-023-03086-6 37872590 PMC10591355

[48] Sun Y Cao Y Song Y Li J Hou Y Huang W . Improved RNA base editing with guide RNAs mimicking highly edited endogenous ADAR substrates. Nat Biotechnol. (2026) 44:464–476. doi: 10.1038/s41587-025-02628-6 40181169

[49] Huang X Lv J Li Y Mao S Li Z Jing Z . Programmable C-to-U RNA editing using the human APOBEC3A deaminase. EMBO J. (2020) 39:e104741. doi: 10.15252/embj.2020104741 33058229 PMC7667879

[50] Kreiter S Vormehr M van de Roemer N Diken M Lower M Diekmann J . Mutant MHC class II epitopes drive therapeutic immune responses to cancer. Nature. (2015) 520:692–6. doi: 10.1038/nature14426 25901682 PMC4838069

[51] Sun Y Wu Y He Z Wang Y Hou W Cao Y . Type III CRISPR-mediated flexible RNA excision with engineered guide RNAs. Mol Cell. (2025) 85:989–998. doi: 10.1016/j.molcel.2025.01.021 39978340

[52] Diaz Quiroz JF Ojha N Shayhidin EE De Silva D Dabney J Lancaster A . Development of a selection assay for small guide RNAs that drive efficient site-directed RNA editing. Nucleic Acids Res. (2023) 51:e41. doi: 10.1093/nar/gkad098 36840708 PMC10123091

