# Supplementary figures and images for: Site-specific neoepitope induction by RNA editing reprograms tumor immunogenicity
Source: Front Immunol. 2026 Jun 9;17:1839930. doi: 10.3389/fimmu.2026.1839930 (PMC13287919; doi:10.3389/fimmu.2026.1839930)

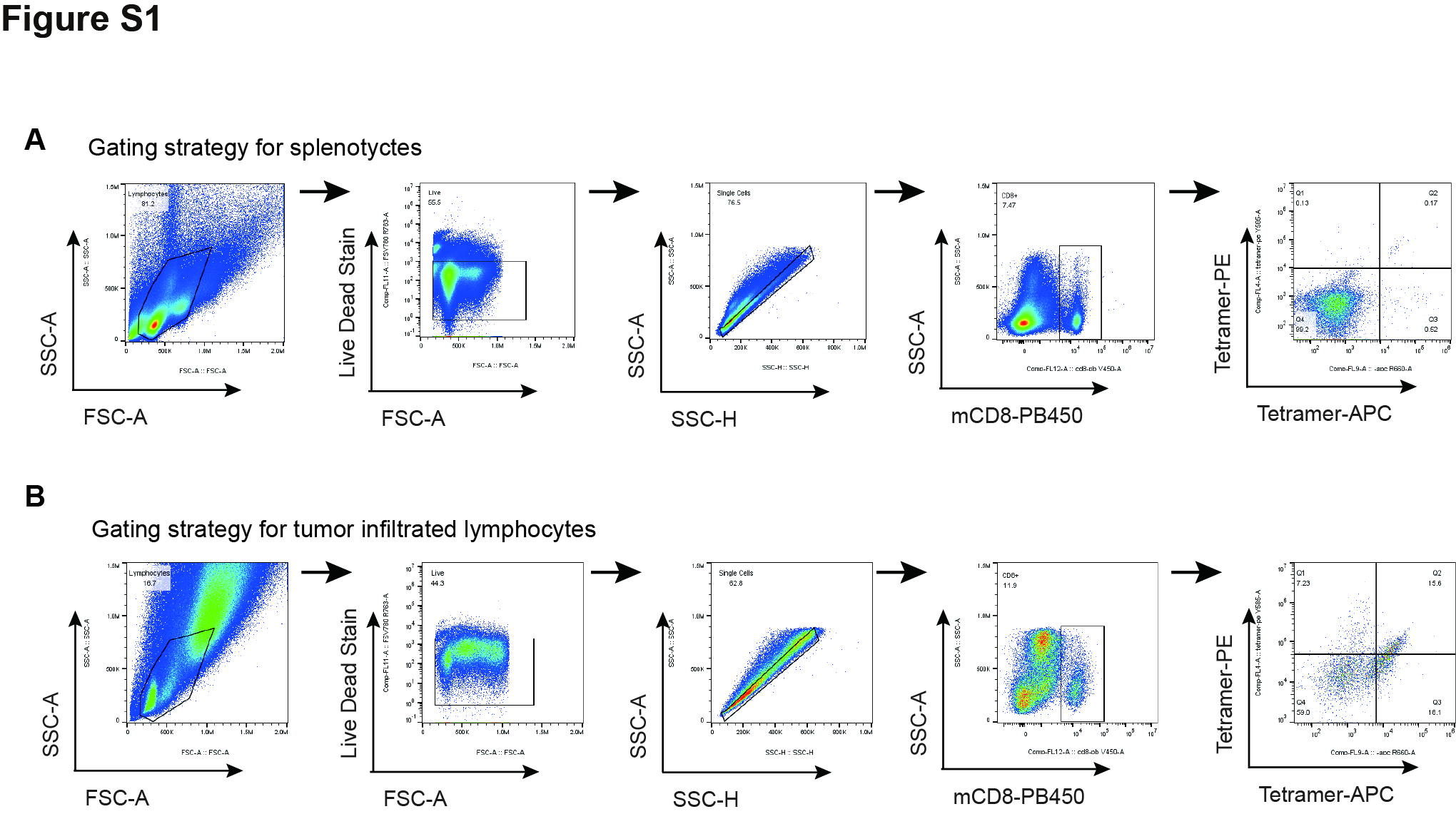

Supplement: Supplementary Figure 1 — Representative gating strategy for splenocytes and tumor infiltrating lymphocytes. (a), Gating strategy for mouse splenocytes. (b), Gating strategy for transplanted mouse tumor-infiltrating lymphocytes. [file Image1.tif]

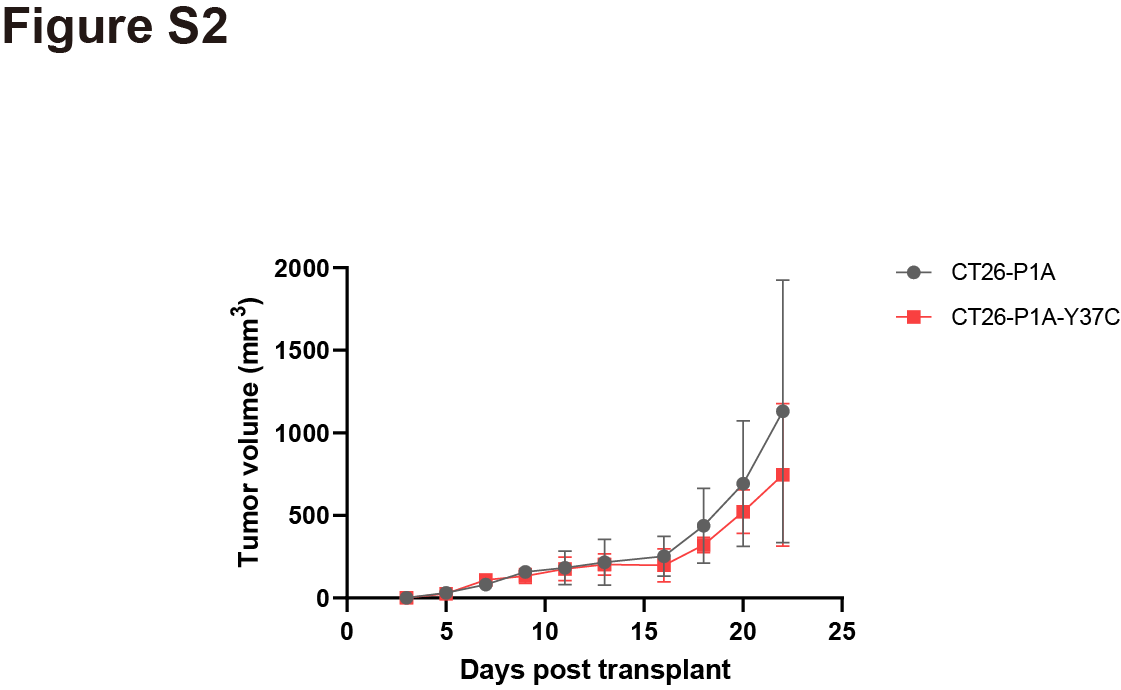

Supplement: Supplementary Figure 2 — P1A-REISN is insufficient to control tumor progression. Female BALB/c mice were transplanted with 2×10^6^ CT26-P1A-Y37C or 1×10^7^ CT26-P1A cells subcutaneously (n = 3 per group). Tumor size was measured every two days. Tumor volume is shown. Data are represented as mean ± SD. [file Image2.tif]

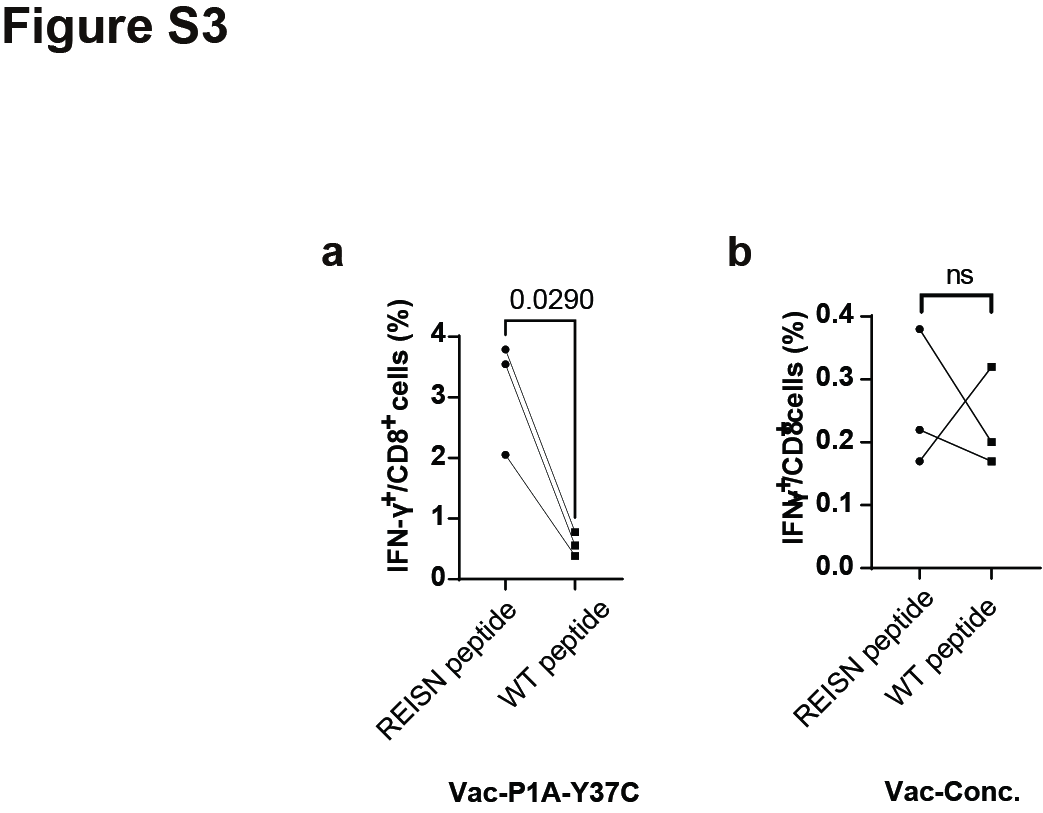

Supplement: Supplementary Figure 3 — Vac-P1A-Y37C circRNA vaccine generate REISN-specific T cell response. (a, b), Quantitation of intracellular cytokine staining of splenocytes from Vac-P1A-Y37C circRNA vaccine (a) and control group (b) stimulated with P1A-REISN peptide and P1A-WT peptide for 12 hours. Paired comparison of T cell responses to P1A-REISN versus P1A-WT peptides were shown. Statistical significance was determined by paired t-test. [file Image3.tif]

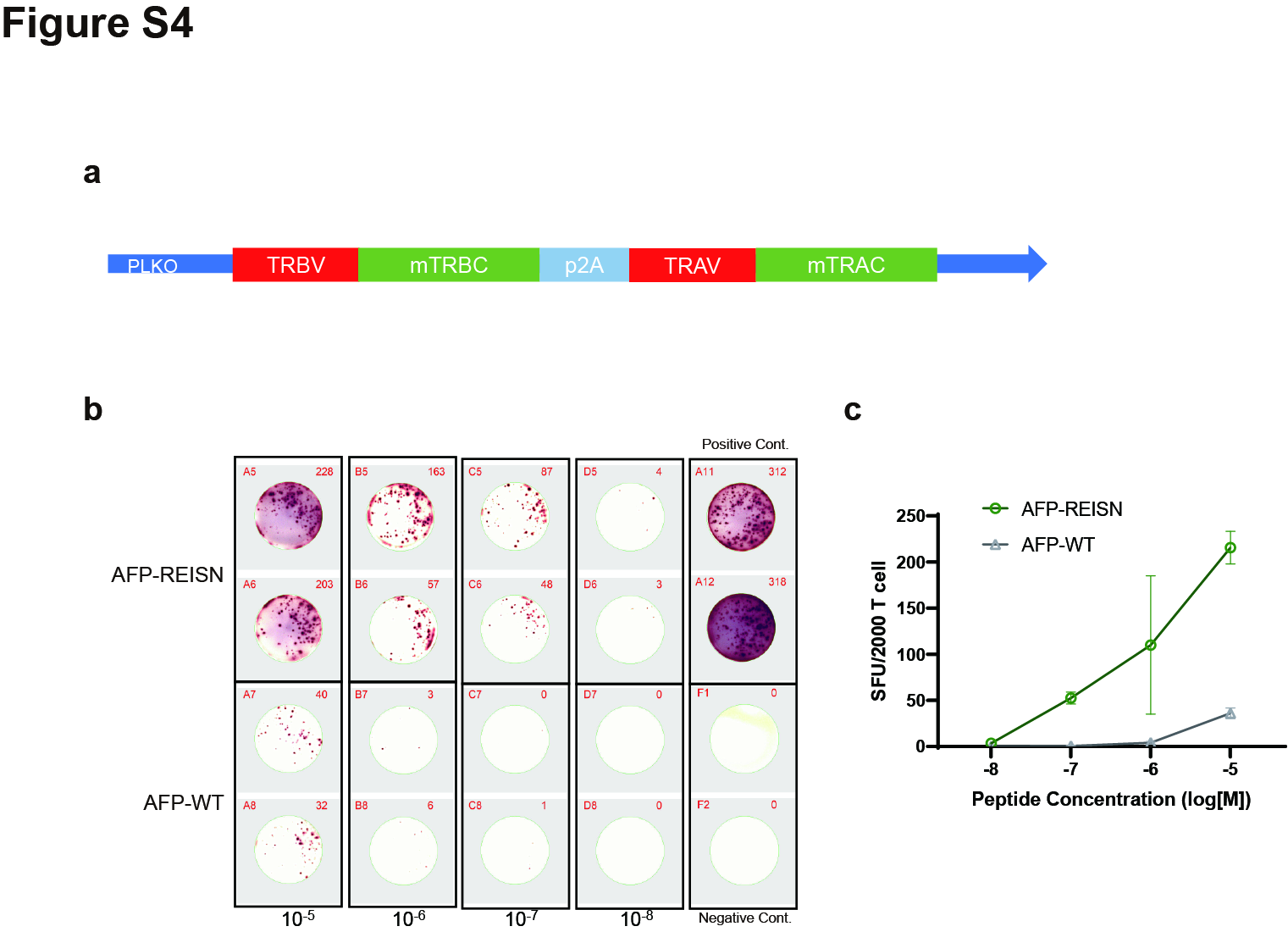

Supplement: Supplementary Figure 4 — TCR041 T cell receptor mediates specific recognition of the AFP-REISN neoantigen. (a), Schematic of TCR041 lentiviral expression vector assembly. (b), Representative ELISpot assay detecting IFN-γ secretion by TCR041-engineered T cells co-cultured with T2 cells pulsed with AFP-REISN or AFP-WT peptides (10 μg/mL, 24 hours). Negative control: T2 cells without peptide. Positive control: PMA and ionomycin. Spot-forming units (SFUs) reflect antigen-specific T cell activation. (c), Quantification of IFN-γ+ SFUs (n = 2 biologically independent experiments). Data are represented as mean ± SD. [file Image4.tif]

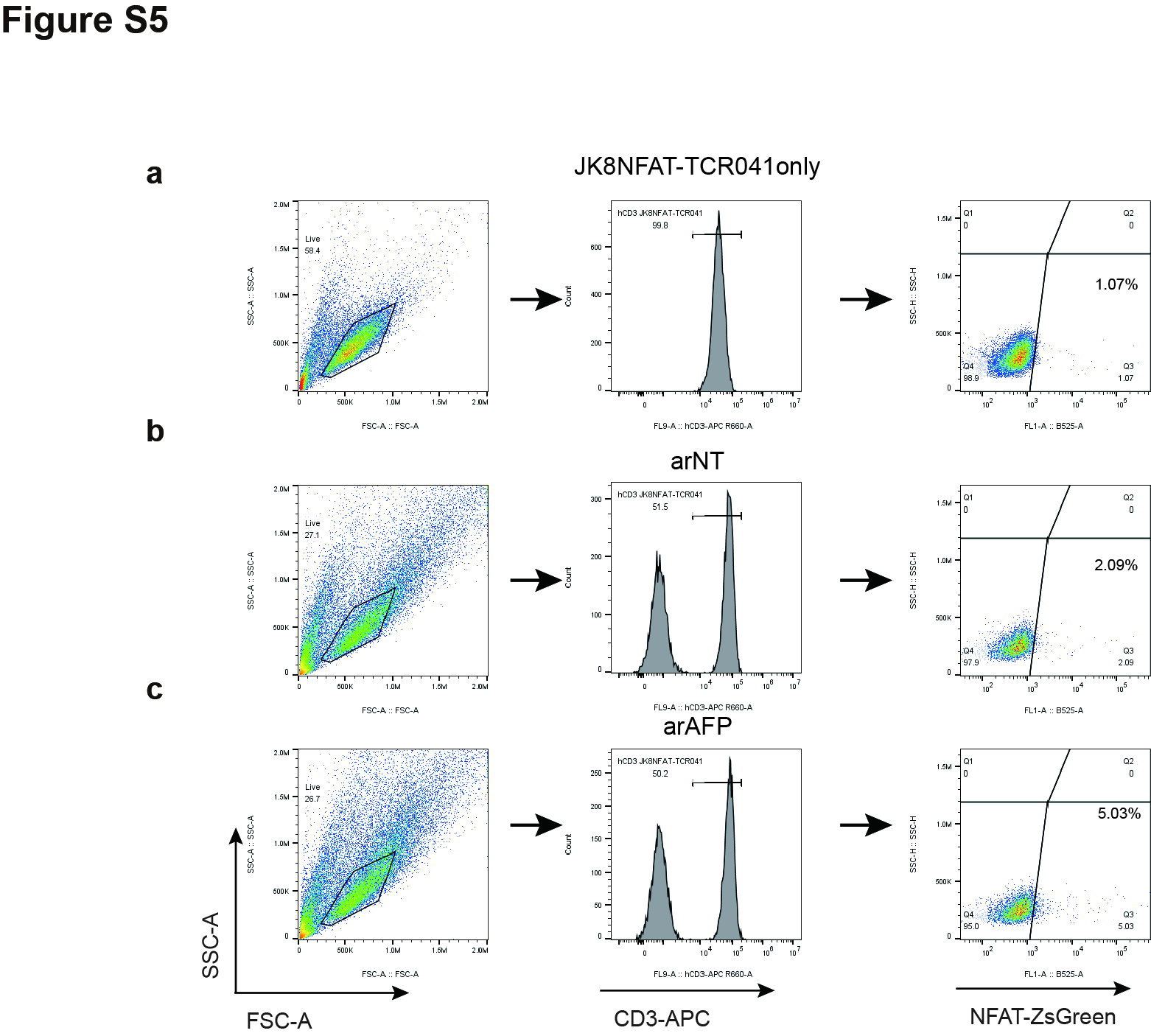

Supplement: Supplementary Figure 5 — JK8NFAT-TCR041 cells detect AFP-REISN2 epitopes generated de novo in 293T cells through transient RNA editing. Representative flow cytometry plots of ZsGreen expression levels in JK8NFAT-TCR041 effector cells only (a), co-cultured with 293T cells transiently transfected with plasmids encoding AFP, MCP-ADAR1, and arNT (b) or arAFP (c) [file Image5.tif]
