# Supplementary material for: Site-specific neoepitope induction by RNA editing reprograms tumor immunogenicity
Source: Front Immunol. 2026 Jun 9;17:1839930. doi: 10.3389/fimmu.2026.1839930 (PMC13287919; doi:10.3389/fimmu.2026.1839930)
Supplement: Supplementary file 6 [file Table1.docx]

**Supplementary tables:**

| **Table S1. Primers and arRNAs** | |
| --- | --- |
| Name | Sequence （5’-3’） |
| arP1A | ctagacaaggcagaatttctt |
| arScramble | ctgcccagagttgttctccat |
| arAFP-1 | tgaatctgttcatgaatgtct |
| arAFP-2 | ataaacgaatttgttcatgaa |
| arMAGE-A1 | tcacacactcaaggactttca |
| arMAGE-A10 | catcacacagccccatcatat |
| arPRAME | cccgacgaggtgctgcaggag |
| arSSX2 | agattctctccgaggctttca |
| arSPAG9-1 | gggcgctgtacttcaggttaa |
| arSPAG9-2 | actgacatttgatctttgtac |
| MAGEA1-RTF1 | gggccaagcacctcttgtat |
| MAGEA1-RTR1 | cttggctgcaactcatgctc |
| MAGEA10-RTF1 | ctggccactcctttgtcctt |
| MAGEA10-RTR1 | agggtaggagaagctacctgt |
| PRAME-RTF1 | actcctcctctcccacatcc |
| PRAME-RTR1 | gtggctgctttgttgcttca |
| SSX2-RTF1 | ctcagagtacgcacggtctg |
| SSX2-RTR1 | ctgtgggtccaggcatgttc |
| P1A-1F | tgataacaagaagccagacaaagc |
| P1ARTR2 | ccaggaaattagggtcgtggaag |
| AFPRTF1 | cagccaaagtgaagagggaaga |
| AFPRTR1 | ttttccccatcctgcagacaat |
| MAGEA1-F | catttcaggtgtcgtgagctagccgccaccatgtctcttgagcagaggagtct |
| MAGEA1-R | gttagtagctccgcttccggatccgactccctcttcctcctctctc |
| MAGEA10-F | catttcaggtgtcgtgagctagccgccaccatgcctcgagctccaaagc |
| MAGEA10-R | gttagtagctccgcttccggattcagggtaggagaagctacctg |
| PRAME-F | catttcaggtgtcgtgagctagccgccaccatggaacgaaggcgtttgtg |
| PRAME-R | gttagtagctccgcttccggatccattaggcatgaaacaggggca |
| MS2-AarI-R | aggtggtcgaccacctgccttggacatgggtgatcctcatgttcggtg |
| MS2-AarI-F | aggtggtcgaccacctgccggcaacatgaggatcacccatgtctttttttaagcttg |
| ADAR1-MCP-pcw57F1 | tcgcctggagaattggctagcgccaccatggc |
| ADAR1-MCP-pcw57R1 | gatcctgtacaacgcgtttaatgatgatgatgatgatggtcgacg |
| ADAR1E1088Q-RTF1 | aggacagcgaggacatgg |
| ADAR1E1088Q-RTR1 | tgtacaggtggaagctcacg |

| **Table S2. Antibodies** | | |
| --- | --- | --- |
| **Antibodies** | **Vendor** | **Cat. No.** |
| FITC anti-mouse CD8a | BioLegend | 100705 |
| Pacific BLUE anti-mouse CD8a | BioLegend | 100728 |
| AF700 anti-mouse CD8a | BioLegend | 100730 |
| APC anti-mouse CD8a | BioLegend | 100712 |
| FITC anti-human CD8a | BioLegend | 300906 |
| APC anti-human CD3 | BioLegend | 300412 |
| APC anti-mouse TCR β chain | BioLegend | 109212 |
| PE anti-mouse TCR β chain | BioLegend | 109208 |
| FITC anti-mouse CD45 | BioLegend | 103108 |
| PB450 anti-mouse CD4 | BioLegend | 100427 |
| PE anti-mouse IFN γ | BioLegend | 505808 |
| PE Rat IgG1,K Isotype Ctrl | BioLegend | 400407 |
| PE Streptavidin | BD Biosciences | 554061 |
| APC Streptavidin | BD Biosciences | 5540667 |
| Death FVS780 | BD Biosciences | 565388 |
